# Pharmacogenetics of human ABC transporter ABCC11: new insights into apocrine gland growth and metabolite secretion

**DOI:** 10.3389/fgene.2012.00306

**Published:** 2013-01-02

**Authors:** Toshihisa Ishikawa, Yu Toyoda, Koh-ichiro Yoshiura, Norio Niikawa

**Affiliations:** ^1^Graduate School of Bioscience and Biotechnology, Tokyo Institute of TechnologyYokohama, Japan; ^2^Omics Science Center, RIKEN Yokohama InstituteYokohama, Japan; ^3^Department of Pharmacy, The University of Tokyo Hospital, Faculty of Medicine, The University of TokyoTokyo, Japan; ^4^Department of Human Genetics, Nagasaki University Graduate School of Biomedical SciencesNagasaki, Japan; ^5^The Research Institute of Personalized Health Sciences, Health Sciences University of HokkaidoIshikari-Tobetsu, Japan

**Keywords:** apocrine gland, earwax, axillary osmidrosis, breast cancer, mastopathy, 5-fluorouracil, tamoxifen

## Abstract

Cell secretion is an important physiological process that ensures smooth metabolic activities and tissue repair as well as growth and immunological functions in the body. Apocrine secretion occurs when the secretory process is accomplished with a partial loss of cell cytoplasm. The secretory materials are contained within secretory vesicles and are released during secretion as cytoplasmic fragments into the glandular lumen or interstitial space. The recent finding that the non-synonymous single nucleotide polymorphisms (SNP) 538G > A (rs17822931; Gly180Arg) in the *ABCC11* gene determines the type of earwax in humans has shed light on the novel function of this ABC (ATP-binding cassette) transporter in apocrine glands. The wild-type (Gly180) of ABCC11 is associated with wet-type earwax, axillary osmidrosis, and colostrum secretion from the mammary gland as well as the potential risk of mastopathy. Furthermore, the SNP (538G > A) in the *ABCC11* gene is suggested to be a clinical biomarker for the prediction of chemotherapeutic efficacy. The aim of this review article is to provide an overview on the discovery and characterization of genetic polymorphisms in the human *ABCC11* gene and to explain the impact of *ABCC11* 538G > A on the apocrine phenotype as well as the anthropological aspect of this SNP in the *ABCC11* gene and patients’ response to nucleoside-based chemotherapy.

## INTRODUCTION

ATP-binding cassette (ABC) proteins form one of the largest protein families encoded in the human genome (Dean et al., 2001; Holland et al., 2003). Hitherto more than 48 human ABC protein genes have been identified and sequenced (Klein et al., 1999). It has been reported that mutations of ABC protein genes are causative of several genetic disorders in humans (Dean et al., 2001). Many of the human ABC proteins are involved in membrane transport of drugs, xenobiotics, endogenous substances, or ions, thereby exhibiting a wide spectrum of biological functions (Schinkel and Jonker, 2003). Based on the arrangement of molecular structure components, i.e., nucleotide binding domains and topologies of transmembrane domains, the hitherto reported human ABC proteins have been classified into seven different sub-families (A to G; Klein et al., 1999; Borst and Elferink, 2002; Ishikawa, 2003).

**FIGURE 1:**
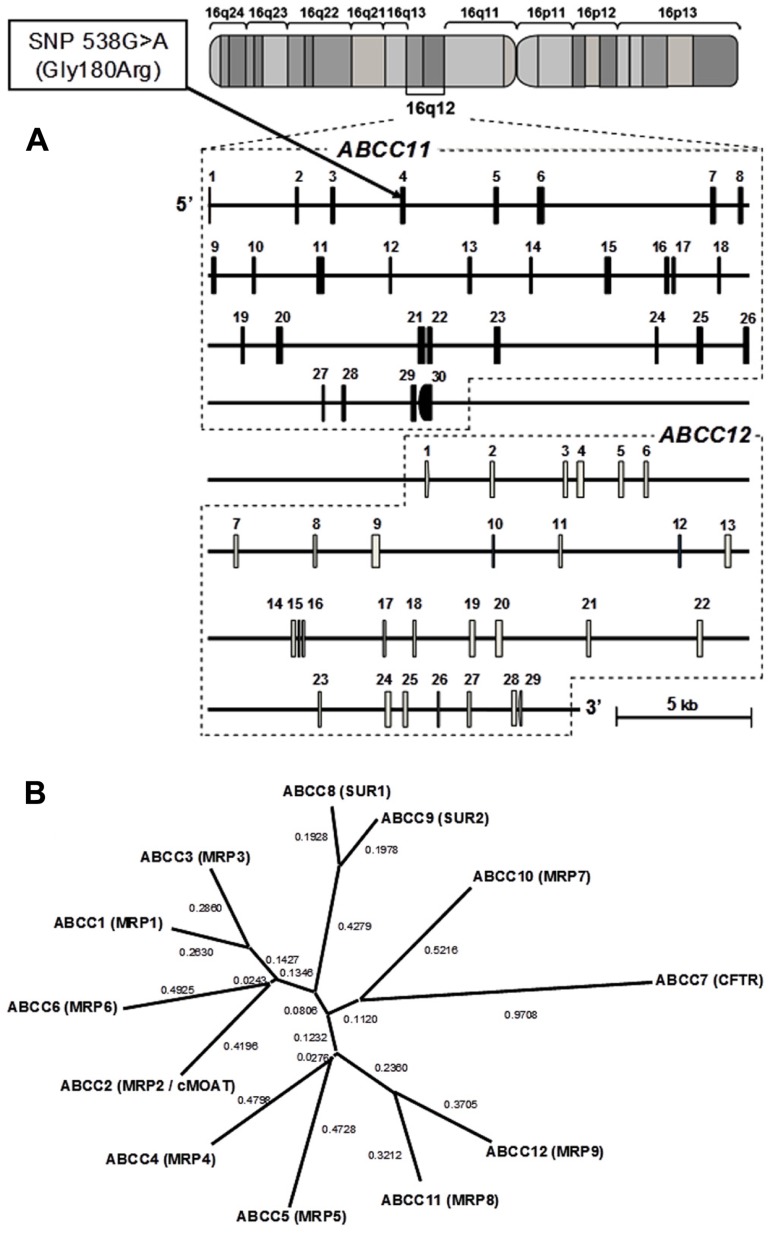
**Schematic illustration of the genomic structures of *ABCC11* and *ABCC12* genes on human chromosome 16q12.1**. **(A)** The cytogenetic location of the *ABCC11* gene as well as the structures of exons and introns were analyzed by BLAST searches on the human genome. A non-synonymous SNP: 538G > A (Gly180Arg), an earwax determinant, is located in exon 4. **(B)** Phylogenetic tree of the ABCC subfamily including CFTR, SUR1, SUR2, and MRPs. The phylogenetic tree was modified from Toyoda et al. (2008). The phylogenetic relationships among members of the “C” sub-family of human ABC transporters were calculated by using the distance-based neighbor-joining methods (Saitou and Nei, 1987).

In 2001, three research groups independently cloned two novel ABC transporters named ABCC11 and ABCC12 from the cDNA library of human adult liver (Bera et al., 2001; Tammur et al., 2001; Yabuuchi et al., 2001). These two genes have been found to be located on human chromosome 16q12.1 in a tail-to-head orientation with a separation distance of about 20 kb (**Figure 1**). The predicted amino acid sequences of both gene products show a high similarity to those of ABCC4 and ABCC5, which suggests that they have the typical structure of “full” ABC transporters with 12 transmembrane helixes and two ABCs. Interestingly, there is no putative mouse or rat orthologous gene corresponding to human *ABCC11* (Shimizu et al., 2003), which indicates that *ABCC11* is not an orthologous gene but rather a paralogous gene generated by gene duplication in the human genome. In contrast, *ABCC12* and its orthologous genes are found in several different species including humans, primates, and rodents (Shimizu et al., 2003; Ono et al., 2007). Transcript analyses suggest that human ABCC11 mRNA is ubiquitously expressed in human adult and fetal tissues (Tammur et al., 2001; Yabuuchi et al., 2001). High levels of ABCC11 mRNA were observed in breast cancer tissues (Bera et al., 2001; Yabuuchi et al., 2001). Table 1 summarizes major findings in the research of the *ABCC11* gene.

**Table 1:** Historical overview on identification of the *ABCC11 *gene and its function.

Year	Scientific progress	Reference
2001	Discovery of human *ABCC11 (MRP8)* gene	Bera et al. (2001), Tammur et al. (2001),Yabuuchi et al. (2001)
2003	Characterization of ABCC11 as a cyclic nucleotide efflux pump	Guo et al. (2003)
2005	*In vitro* characterization of substrate selectivity of ABCC11	Chen et al. (2005)
2006	Characterization as an apical efflux pump for steroid sulfates in CNS	Bortfeld et al. (2006)
2006	Identification of *ABCC11 *SNP c.538G > A as the determinant of human earwax type	Yoshiura et al. (2006)
2007	Association between the degrees of apocrine colostrum secretion and *ABCC11 *genotype	Miura et al. (2007)
2008	Involvement of ABCC11 in 5-fluorouracil resistance in lung cancer cell line	Oguri et al. (2007)
2008	Regulation of ABCC11 expression by estrogen in MCF7 cells	Honorat et al. (2008)
2009	Discovery of ubiquitination and proteasomal degradation of SNP c.538G > A variant	Toyoda et al. (2009)
2009	Association between axillary osmidrosis and ABCC11 wild-type	Nakano et al. (2009), Toyoda et al. (2009),Inoue et al. (2010)
2009	Japanese map of the earwax gene frequency: a nationwide collaborative study	Super Science High School Consortium (2009)
2010	Association between breast cancer risk and *ABCC11 *wild-type in Japanese women	Ota et al. (2010), Toyoda and Ishikawa (2010)
2010	Involvement of ABCC11 in pemetrexed resistance in lung cancer	Uemura et al. (2010)
2011	No association between breast cancer risk and *ABCC11 *wild-type in European women	Beesley et al. (2011), Lang et al. (2011)
2011	Down-regulation of ABCC11 protein in human breast cancer	Sosonkina et al. (2011)

**Figure A1:**
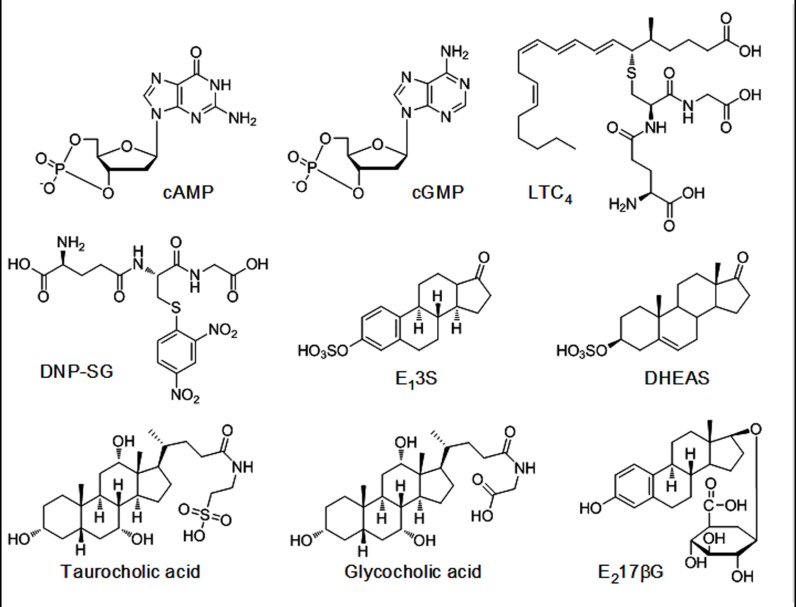
**Chemical structures of ABCC11 substrates**. Cyclic adenosine monophosphate (cAMP), cyclic guanosine monophosphate (cGMP), leukotriene C_4_ (LTC_4_), S-(2,4-dinitrophenyl)-glutathione (DNP-SG), estrone 3-sulfate (E_1_3S), dehydroepiandrosterone 3-sulfate (DHEAS), and E_2_17βG (estradiol 17-β-D-glucuronide).

When transfected exogenously, the ABCC11 wild-type (WT) protein was localized in the apical membrane of Madin–Darby canine kidney cells strain II (MDCK II cells; Bortfeld et al., 2006). The substrate specificity of ABCC11 WT was characterized in more detail by an *in vitro* transport assay with plasma membrane vesicles prepared from pig LLC-PK1 cells transfected with an ABCC11 WT expression vector (Chen et al., 2005). The results of this assay demonstrated that ABCC11 WT is able to transport a variety of lipophilic anions including cyclic nucleotides, glutathione conjugates such as leukotriene C_4_ (LTC_4_) and S-(2,4-dinitrophenyl)-glutathione (DNP-SG), steroid sulfates such as estrone 3-sulfate (E_1_3S) and dehydroepiandrosterone 3-sulfate (DHEAS), glucuronides such as estradiol 17-β-D-glucuronide (E_2_17βG), the monoanionic bile acids glycocholate and taurocholate, as well as folic acid and its analog methotrexate (MTX; Chen et al., 2005; Bortfeld et al., 2006). Chemical structures of these compounds are presented in **Figure A1** in Appendix. While ABCC11 transports a variety of organic anions, endogenous natural substrates for this transporter have not yet been identified.

## GENETIC POLYMORPHISMS AND PHYSIOLOGICAL FUNCTION OF ABCC11

**FIGURE 2:**
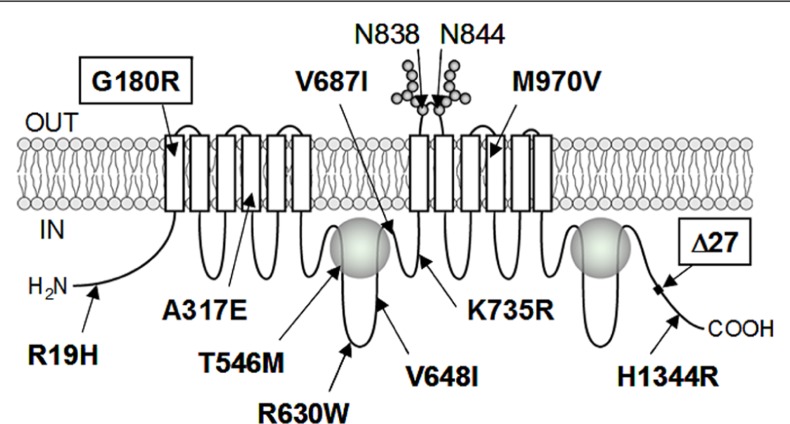
**Schematic illustration of ABCC11 protein structure and hitherto known non-synonymous SNPs**. ABCC11 has a total of 12 transmembrane (TM) regions and two intracellular ATP-binding cassettes. Asn838 and Asn844 residing in an extracellular loop between transmembrane helices TM7 and TM8 are *N*-linked glycosylation sites in the ABCC11 WT protein. Locations of hitherto reported non-synonymous SNPs and ∆27 (a rare deletion mutation) are indicated in the putative structure of ABCC11. G180R and ∆27 are related to the formation of dry-type earwax.

**FIGURE 3:**
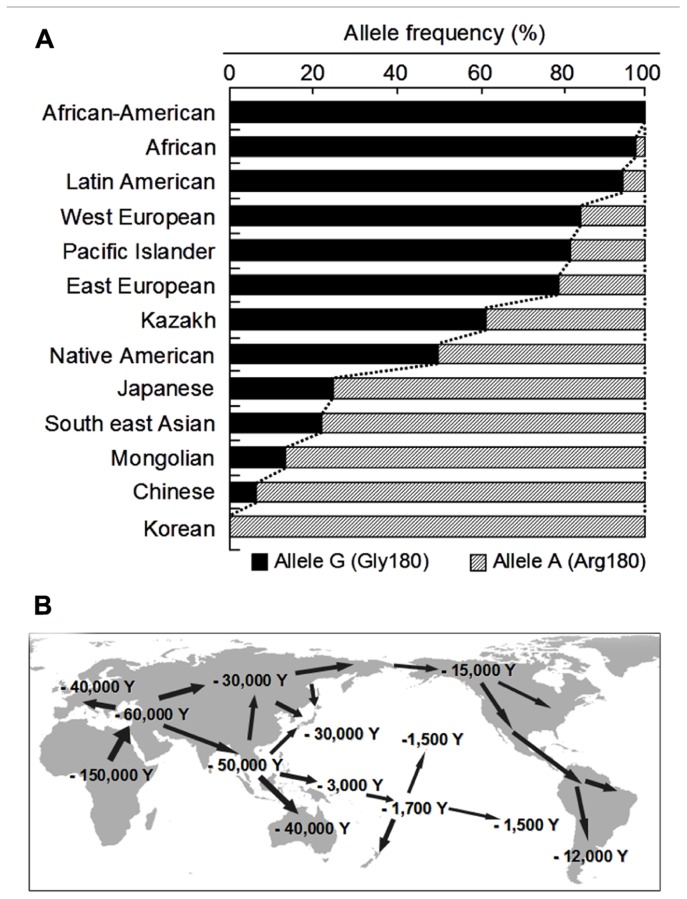
**The allele frequencies of the wild-type (WT; Gly180) and 538G > A (Arg180) variant of human *ABCC11* among different ethnic populations **(A)** and inter-continental migration of *Homo sapiens***(B)****. Data are from Yoshiura et al. (2006) and Toyoda et al. (2008).

To date, more than 10 non-synonymous single-nucleotide polymorphisms (SNPs) have been reported in the human *ABCC11* gene (**Figure 2**). Among those SNPs, one SNP (rs17822931; 538G > A, Gly180Arg) determines the human earwax type (Yoshiura et al., 2006). Interestingly, this SNP (538G > A) exhibits wide ethnic differences in allelic frequency (Table 2). In Mongoloid populations in Asia, the frequency of the 538A allele is predominantly high, whereas the frequency of this allele is low among Caucasians and Africans (Yoshiura et al., 2006; Toyoda et al., 2008; **Figure 3A**). The frequency of the 538A allele exhibits a north-south and east-west downward geographical gradient with the highest peak in Korea. It is suggested that the 538A allele arose in northeast Asia and thereafter spread throughout the world (Yoshiura et al., 2006), apparently reflecting the inter-continental migration of *Homo sapiens* (**Figure 3B**). A similar geographical gradient was also observed in the frequency of the 2677G (Ala893) allele of the *ABCB1* (*P-glycoprotein*/*MDR1*) gene (Sakurai et al., 2007). In this regard, anthropological aspects of SNP 538G > A in the *ABCC11* gene are described in the following section.

**Table 2:** Frequencies of *ABCC11 *genotypes and allele c.538A among different ethnic groups.

Ethnic groups	Tribes or inhabitants	No. of individuals with genotypes			Number of individuals genotyped	Frequency of allele“A”
		AA (frequency)	GA	GG		
Korean^§^	Daegu city inhabitants	100 (1.000)	0	0	100	1.000
Chinese^§^	Northern and southern Han Chinese	42 (0.808)	10	0	52	0.904
Mongolian	Khalkha tribe^§^	126 (0.759)	36	4	166	0.867
Japanese	Nagasaki people (West-end prefecture of Japan mainland)^§^	87 (0.690)	35*	4	126	0.829
	Okinawa people (Southwest-end prefecture of Japan)	30 (0.517)	25	3	58	0.733
	Yonaguni islander (West-end island of Japan)^§^	13 (0.433)	15	2	30	0.683
	Ainu in Biratori-Nibutani village in Hokkaido^§^	31 (0.534)	23	2	58	0.733
Vietnamese	People from multiple regions	82 (0.536)	60	11	153	0.732
Thai	Northern Thai [Lahu, Shan, Lisu, Hmong, Akha, Mlaburi, and Karen (Mae-sot Thai) tribes combined]	215 (0.505)	163	48	426	0.696
	Central Thai in Bangkok	31 (0.633)	10	8	49	0.735
	Southern Thai (Orang Laut and Sakai tribes combined)	2 (0.026)	23	52	77	0.175
Indonesian	Dayak tribe in Kalimantan	12 (0.293)	23	9	41	0.573
	Toraja and Bugis tribes in Sulawesi	27 (0.270)	49	24	100	0.515
	Flores	18 (0.300)	25	17	60	0.508
	Sumba	9 (0.180)	16	25	50	0.340
	WA tribe in Irian Jaya	0 (0.000)	2	31	33	0.030
Malaysian	Sabah in North Borneo^§^	24 (0.393)	27	10	61	0.566
	Bentong tribe	8 (0.113)	40	23	71	0.394
Taiwanese	Taiwan Aborigine (Yami and Ami combined)	34 (0.330)	48	21	103	0.563
Native American^§^		6^†^ (0.300)	8^†^	6	20	0.500
Filipino	Palawan	11 (0.229)	23	14	48	0.469
Easter Islander^§^		4 (0.148)	17	6	27	0.463
Pacific islander^§^		1 (0.143)	1	5	7	0.429
Bolivian^§¶^	Aymara	5 (0.167)	14	11	30	0.400
Kazakh		6 (0.200)	11	13	30	0.383
Native Paraguayan	Ayoreos	2 (0.040)	34	14	50	0.380
	Sanapana	0 (0.000)	14	61	75	0.093
Russian^§^		5 (0.045)	45	62	112	0.246
Solomon Islander^§^		2 (0.323)	25	35	62	0.234
French^§^	From the CEPH families	1 (0.083)	3	8	12	0.208
Andes people^§^		1 (0.100)	2^†^	6	10	0.200
Hungarian^§^		0 (0.000)	4	6	10	0.200
Jew^§^	Ashkenazi	0 (0.000)	4	6	10	0.200
Ukrainian^§^		0 (0.000)	15	27	42	0.179
Papuan	Papua New Guinea	1 (0.026)	11	26	38	0.171
American of European	From CEPH families without the French and Venezuelans ancestry^§^	1 (0.012)	16	65	82	0.110
Venezuelan^§^	Ye’Kuana village	0 (0.000)	3	11	14	0.107
	Sanuma village	0 (0.000)	0	19	19	0.000
Vanuatu islander	Aneityum and Santo islanders combined	1 (0.011)	17	74	92	0.103
Iberian^§^		0 (0.000)	2	8	10	0.100
Columbian		0 (0.000)	2	17	19	0.053
African	From various sub-Saharan nations	0 (0.000)	1	11	12	0.042
American of African ancestry^§^		0 (0.000)	0	10	10	0.000

*Data are from Yoshiura et al. (2006).*

§ Examined for a 27-bp deletion (A27) in *ABCC11*;

* One e xceptional case of dry cerumen who has the deletion;

† One each case of the deletion;

¶ Nine cases of the deletion.

Earwax (cerumen) is a secretory product of the ceruminous apocrine glands, which can be classified into two phenotypes in humans, wet (sticky) and dry. The dry-type is most commonly found within the Asian population, especially among Koreans, Japanese, and Chinese; whereas the wet-type is the dominant phenotype for many Africans and Caucasians. The 538A/A genotype gives the dry phenotype, whereas both the 538G/A and G/G genotypes give the wet phenotype. This relationship is consistent with observations that earwax type is a Mendelian trait and that the wet phenotype is dominant to the dry one.

Immunohistochemical studies with cerumen gland-containing tissue specimens revealed that the ABCC11 WT protein with Gly180 was expressed in the cerumen gland (Toyoda et al., 2009). The cerumen gland is one of the apocrine glands. In addition to their presence in the external auditory canal, apocrine glands can be found in the axillary region and breast, whose physical characteristics also are concerned with apocrine glands. In fact, there is a positive association among the wet earwax type, axillary osmidrosis (Yoo et al., 2006), and colostrum secretion from the breast (Miura et al., 2007).

## ANTHROPOLOGICAL ASPECTS OF ALLELES 538G AND 538A

It is generally accepted that, since they migrated out-of-Africa, humans spread all over the world with great diversity (Cavalli-Sforza, 2005). Whilst the routes of migration followed by the ancient Mongoloid people remain obscure, two different routes have been proposed (**Figure 3B**). After branching from a main stream common to the ancient Caucasoids 150,000–60,000 years ago (Nei, 1982), the ancient Mongoloids migrated to Southern Asia. It is assumed that one branch remained there or further migrated more south-eastwardly through the so-called Sundaland and Sahuland, and finally reached the Australian continent 50,000–46,000 years ago (Bowler et al., 2003). The last wave of migration of people to the Southern Pacific islands took place more recently, 3,000–1,500 years ago. On the other hand, another branch migrated northward and reached an area around Lake Bikal of Siberia along the Altai mountains. Alternatively, a branch of the ancient Mongoloids might have directly migrated to Siberia from the common stream or migrated from South East Asia toward North East Asia (The HUGO Pan-Asian SNP Consortium, 2009). As an expansion of the last glaciation occurred 30,000–15,000 years ago, small tribes of the ancient Northern Mongoloids may have long been isolated by the glaciers.

As described above, wet/dry types of earwax are determined by the SNP c.538G > A in the *ABCC11* gene; G/A and G/G genotypes give the wet-type and A/A the dry-type. There is a hypothesis that a c.538G (wet-type) → c.538A (dry-type) mutation may have occurred some 40,000 years ago in a tribe of the ancient Northern Mongoloids (Ohashi et al., 2011). Subsequent spreading of the dry-type among the Mongoloids may be explained by a certain selective advantage of the mutation (Matsunaga, 1962), or dry cerumen might have been evolutionally neutral, which would have led to its spread as an attribute of genetic drift. Based on the geographical gradient distribution of the 538A allele with a peak in East Asia, especially in Northern China and Korea, one might assume that the c.538G → c.538A mutation arose somewhere in Siberia. The north-south downward gradient of the 538G allele from Northern China toward Japan and Southern Asia might reflect the Ancient Northern Mongoloid migrations. Similarly, the east-west gradient from Siberia toward Europe (Spitsyn and Afanasèva, 1989) is partly the consequence of Mongolian migration, especially as their invasions pushed westward from about A.D. 500 until A.D. 1500. The situation is much like the frequency of type B for the ABO blood group, which is high in Asia (>25%) and low (<10%) in Europe (Roychoudhury and Nei, 1988). Similar geographic distribution and ethnic differences are known for the *ALDH2* gene, one of the major determinants for alcohol sensitivity (Goedde et al., 1992; Luo et al., 2009).

The relatively high frequency of the 538A allele among present-day Native Americans suggests that their ancestors may have undertaken long journeys from Siberia through the Bering land-bridge (Beringia) to the American continent during the past 15,000 years (Horai et al., 1993; Bonatto and Salzano, 1997; Tokunaga et al., 2001; Dillehay, 2003). Based on recent craniometric studies of skeletons from archeological remains in the Baja California peninsula, however, it has also been postulated that an earlier migration wave from that via the northern route might have occurred from islands of south-eastern Asia by an ancestor common to both Palaeoamericans and Australians around 40,000–12,000 years ago (Gonzáles-Josè et al., 2003). Furthermore, the allelic data for South Americans revealing the 538A allele frequency of 0.400 in Bolivia, 0.200 in the Andes region, 0.093–0.380 in Paraguay, 0.053 in Colombia, and 0.000 in Venezuela rather favors a hypothesis of an ancient migration through a “pacific coast road” along the Andes mountain range.

## ROUTES OF THE JAPANESE POPULATION

The Japanese population is considered to have a dual structure comprising descendants of mixtures between the ancient “Jomon” and “Yayoi” populations. The term “Jomon” is derived from characteristic twisted cord striations or marks on earthenware used during a prehistoric time (13,000–3,000 years ago) in Japan. As it has been reported that the Jomon had occupied various areas of Japan prior to the Yayoi’s appearance, they apparently were either assimilated or rather moved away from the Yayoi (Iizuka and Nakahashi, 2002; Temple et al., 2008; Temple, 2010). It is hypothesized that the dry earwax type was introduced by the Yayoi people to the Jomon population, where the wet-type had been predominant.

Since the admixture of the two Jomon and Yayoi populations is still not complete in Japan even now, the 538G allele frequency is higher in the rather remote areas where the Jomon moved away from the Yayoi’s peopling route within the Japanese islands. The Ainu-Japanese people living in a Japanese northern island “Hokkaido” are aboriginal inhabitants of Japan. Based on morphological and mitochondrial DNA polymorphism studies, it has been hypothesized that both the Ainu- and the Okinawa-Japanese living in a Japanese southern island “Okinawa” are descendants of the ancient native Japanese, “Jomon” people (Horai et al., 1991). Molecular studies demonstrated that Ainu-Japanese still retain a certain degree of their own genetic uniqueness among surrounding populations, and exhibit considerable genetic distance from other East Asian populations (Tokunaga et al., 2001; Tajima et al., 2004). As far as wet-type of earwax and the 538G allele frequency are concerned, the Ainu- and Okinawa-Japanese people are not the direct descendants “Yayoi” from the Ancient Northern Mongoloids of Siberian origin.

To analyze the nationwide allele frequency, the Super Science High School (SSH) consortium collected a total of 1963 fingernail samples of pupils/students from at least one high school/university in every prefecture in Japan (Super Science High School Consortium, 2009). Although the 538G allele frequency varied among the 47 prefectures, the Gifu/Kyoto and Okinawa prefectures showed the lowest and highest values for the 538G allele, respectively. Other areas with low frequencies of the 538G allele included Northeastern Kyushu, Northern Shikoku, and Kinki districts, showing a belt-like zone, whereas those with high frequencies of the 538G allele next to Okinawa were the Southwestern Kyushu, Hiroshima prefecture, and Tohoku districts. Those observations strongly suggest that the admixture of “Jomon” and “Yayoi” populations is still not complete in Japan.

## STRONG ASSOCIATION BETWEEN AXILLARY OSMIDROSIS AND THE GENOTYPE OF *ABCC11* 538G > A

Today in Japan, axillary osmidrosis is recognized as a disease that is covered by the national health insurance system. Axillary osmidrosis, which is exemplified by unpleasant odors, sweating and staining of clothes, is often perceived, especially by young women, as a distressing and troublesome problem (Wu et al., 1994). Axillary osmidrosis is a chronic skin condition characterized by an excessive, axillary malodor resulting from apocrine gland dysfunction (Hess et al., 2008). Certain people display an excessive fear, aversion or psychological hypersensitivity to unpleasant smells or odors. They tend to opt for aggressive surgical treatments and are sometimes categorized as having osmophobia. Interestingly, an association between wet-type earwax and axillary osmidrosis had already been recognized more than half a century ago (Matsunaga, 1962). Hence, the wet-type of earwax has frequently been used as one of diagnostic criteria and characteristics in the clinic. This relationship, however, had only been based on the observations of those two respective phenotypes. Therefore, there has been a need for objective evidence for diagnosis of axillary osmidrosis to prevent unnecessary treatments for such patients.

Recently, it has been reported that the ABCC11 WT allele is intimately associated with axillary osmidrosis as well as the wet-type of earwax (Table 3). Several studies have already concluded that the genotypes at *ABCC11* 538G > A would be useful biomarkers for the diagnosis of axillary osmidrosis (Nakano et al., 2009; Toyoda et al., 2009; Inoue et al., 2010; Martin et al., 2010). Therefore, it is suggested that genotyping of the *ABCC11* gene would provide an accurate and practical criterion for guidance of appropriate treatment and psychological management of patients (Toyoda et al., 2009; Inoue et al., 2010; Ishikawa and Hayashizaki, 2012). Rapid genotyping of the *ABCC11* gene is briefly described in Appendix.

**Table 3:** Association of *ABCC11* genotype with earwax type and axillary osmidrosis in Japanese subjects.

Earwax type	Genotype at *ABCC11* 538G > A			Axillary osmidrosis patients
		G/G	G/A	A/A	
Dry	262	0	0	262	0
Wet	300	23	277	0	182

*Data are calculated from Inoue et al. (2010) and Nakano et al. (2009)*.

Sweat produced by the axillary apocrine glands is odorless. Secretions from the apocrine glands, however, can be converted to odoriferous compounds by bacteria (*Corynebacteria*), which results in the formation of the unique “human axillary odor” (Shehadeh and Kligman, 1963). Axillary osmidrosis patients (538G/G homozygote or G/A heterozygote) were observed to have significantly more numerous and larger-sized axillary apocrine glands as compared with those in subjects carrying the A/A homozygote. Indeed, the 538G allele in the *ABCC11* gene is associated with axillary osmidrosis (Nakano et al., 2009; Toyoda et al., 2009; Inoue et al., 2010; Martin et al., 2010), and ABCC11 WT (Gly180) would be responsible for the secretion of preodoriferous compounds from the axillary apocrine gland. In primates, axillary odors may play a role in olfactory communication, although no documented behavioral or endocrine changes resulting from volatiles produced in the axillae have been reported to occur in humans. Previous studies have described the presence of androgen steroids in the axillary area. Androsterone sulfate (AS) and DHEAS were detected in an extract of axillary hairs in addition to high levels of cholesterol (Julesz, 1968). It was also demonstrated, following injection of radioactive pregnenolone or progesterone, that steroid secretion was concentrated in the axillary area (Brooksbank, 1970). In those studies, however, the axillary sweat collected from the skin surface contained a mixture of materials from apocrine, eccrine, and sebaceous glands, in addition to desquamating epidermal cells. In this respect, Labows et al. (1979) demonstrated that pure apocrine secretions contained at least two androgen steroids, AS and DHEAS, in addition to cholesterol. It is strongly suggested that one of the physiological functions of ABCC11 WT is the active transport of steroid metabolites, such as AS and DHEAS, into the lumen of apocrine glands.

## ENDOPLASMIC RETICULUM-ASSOCIATED DEGRADATION OF THE SNP VARIANT OF ABCC11

Why does one SNP (c.538G > A) in the human *ABCC11* gene affect the function of apocrine glands? To address this question, we have recently provided evidence that proteasomal degradation of the SNP variant (Arg180) of ABCC11 is the underlying molecular mechanism (Toyoda et al., 2009). ABCC11 WT with Gly180 is an *N*-linked glycosylated protein, which is localized within intracellular granules and large vacuoles as well as at the luminal membrane of secretory cells in the cerumen apocrine gland (Toyoda et al., 2009). *N*-linked glycosylation occurs at both Asn838 and Asn844 in the extracellular loop between transmembrane domains 7 (TM7) and 8 (TM8) of the ABCC11 WT protein. In contrast, the SNP variant (Arg180) lacks *N*-linked glycosylation and readily undergoes proteasomal degradation, most probably via ubiquitination. As a consequence, no granular or vacuolar localization was detected in the cerumen apocrine glands of people homozygous for the SNP variant.

Morphological differences were previously reported between the secretory cells of wet and dry types of human ceruminous glands (Shugyo et al., 1988). In the wet-type glands, the Golgi apparatus was reportedly well developed, whereas it was generally small in the corresponding cells of the dry-type. Furthermore, numerous intracellular granules were observed in the wet-type gland in close relationship to their well-developed Golgi apparatus, whereas intracellular granules were rare in the dry-type gland.

**FIGURE 4:**
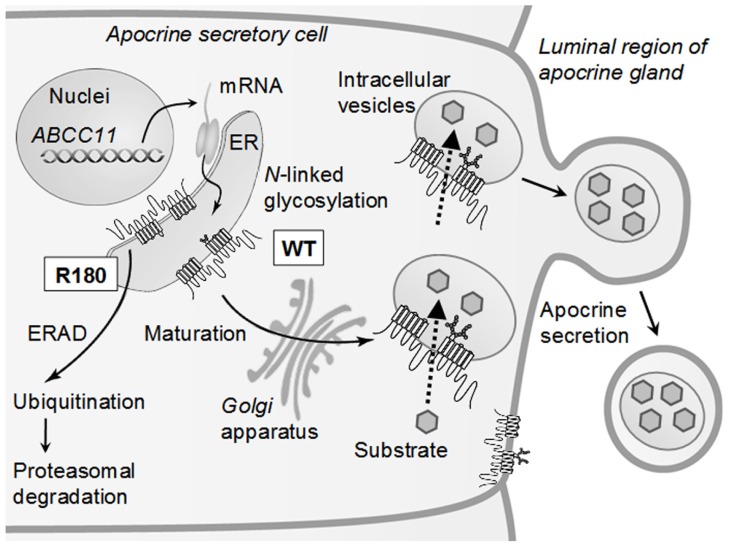
**Schematic illustration of intracellular sorting of ABCC11 WT and proteasomal degradation of the R180 (Arg180) variant in secretory cells of the apocrine gland**. *De novo* synthesized ABCC11 WT is *N*-linked glycosylated at Asn838 and Asn844 in the ER, further processed in the Golgi apparatus, and destined for the membranes of intracellular granules and vacuoles. Ceruminous components are thought to be transported by ABCC11 WT and sequestered in intracellular granules and vacuoles. SNP variant R180 lacking *N*-linked glycosylation is recognized as a misfolded protein in the ER and readily undergoes ubiquitination and proteasomal degradation (ERAD pathway). ER, endoplasmic reticulum; ERAD, ER-associated degradation. This scheme is modified from Toyoda et al. (2009).

The endoplasmic reticulum (ER) and Golgi apparatus are the synthesis and maturation sites of proteins destined for the plasma membrane, the secretory and endocytic organelles, and secretion (Ellgaard et al., 1999; Helenius and Aebi, 2004). Efficient quality control systems have evolved to prevent incompletely folded proteins from moving along the secretory pathway. Accumulation of misfolded proteins in the ER would detrimentally affect cellular functions. Therefore, misfolded proteins may be removed from the ER by retrotranslocation to the cytosol compartment where they are degraded by the ubiquitin-proteasome system. This process is known as ER-associated degradation (ERAD; Mori, 2000; Ellgaard and Helenius, 2001; Hampton, 2002; Kleizen and Braakman, 2004). It is likely that the product of the SNP variant (Arg180) is recognized as a misfolded protein in the ER and readily undergoes proteasomal degradation. An electrostatic charge (either positive or negative) at amino acid 180 in the transmembrane domain 1 (TM1) might interfere with correct folding of the *de novo* synthesized ABCC11 protein in the ER (Toyoda et al., 2009). This ERAD processing of the SNP variant (Arg180) of ABCC11 may greatly influence the activity of ceruminous apocrine glands and determine the type of human earwax. Similar ERAD processing is considered to take place for the SNP variant (Arg180) of ABCC11 in axillary and mammary apocrine glands. **Figure 4** schematically illustrates the impact of this SNP on the cellular localization and function of ABCC11 in secretory cells of the apocrine gland. Asn838 and Asn844 are glycosylation target sites in the human ABCC11. The *N*-linked glycans are thought to be subjected to extensive modification as glycoproteins mature and move through the ER via the Golgi apparatus to their final destinations as, for example, intracellular granules and large vacuoles of secretory cells in the apocrine gland.

## ABCC11 WILD-TYPE ALLELE AND BREAST CANCER RISK

In 1971, Petrakis (1971) first reported that international mortality and frequency rates for breast cancer seemed to be associated with the frequency of the allele for wet-type earwax. Caucasians and African–Americans in the USA as well as Germans exhibited approximately fourfold higher rates of breast cancer mortality as compared with Japanese and Taiwanese women (Petrakis, 1971). Nevertheless, the phenotypic association of the wet-type of earwax with breast cancer remained controversial (Petrakis, 1971; Ing et al., 1973).

At the present time, it is not well understood whether ABCC11 WT really contributes to breast cancer risk. Therefore, we have most recently carried out a genotyping study of the SNP 538G > A (Gly180Arg) for a total of 543 Japanese women to examine the association between the frequency rate of breast cancer and the allelic frequency of the G allele (WT). We obtained blood samples from patients with invasive breast cancer (*n* = 270) and control volunteers (*n* = 273) and genotyped the SNP c.538G > A in the *ABCC11* gene. The frequency of the 538G allele in the breast cancer patients was higher than that in the control volunteers. The odds ratio for the women with genotypes (G/G + G/A) to develop breast cancer was estimated as 1.63 (*p*-value = 0.026), suggesting that the 538G allele in the *ABCC11* gene is moderately associated with the risk of breast cancer (Ota et al., 2010). The relative ratio of breast cancer patients carrying the homozygous 538G/G allele was 1.77-fold greater than that of the corresponding healthy volunteers (Ota et al., 2010). This relative ratio was even greater than that (1.41-fold) for breast cancer patients carrying heterozygous alleles 538G/A. The G allele appears to be positively related to breast cancer frequency in the groups of Japanese women studied. In contrast, no significant association with breast cancer risk was observed in Europeans (Beesley et al., 2011; Lang et al., 2011).

**FIGURE 5:**
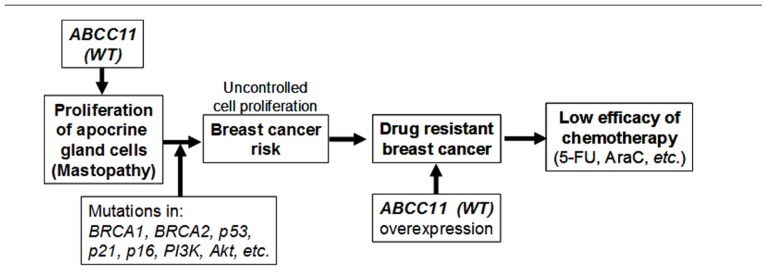
**The potential impact of* ABCC11 WT* (538G) on the apocrine phenotype, patients’ response to nucleoside-based chemotherapy, and the potential risk of mastopathy and breast cancer**. BRCA-1, breast cancer-1; BRCA-2, breast cancer-2; PI3K, phosphatidylinositol 3-kinase; ERα (+), estrogen receptor α-positive; 5-FU, 5-fluorouracil; AraC, cytarabine. This scheme is modified form Toyoda and Ishikawa (2010).

We initially thought that some genetically determined variation(s) in the apocrine system might influence susceptibility to breast cancer, although the genetic determinant (538G > A SNP in *ABCC11*) was not known at that time. It is hypothesized that the function of ABCC11 *per se*, or metabolites transported by ABCC11, may stimulate the proliferation of apocrine gland cells to enhance the risk of mastopathy (**Figure 5**). This hypothesis is supported by evidence that apocrine glands are large in individuals carrying the WT allele of the *ABCC11* gene. So far as the cell cycle machinery is operating normally, proliferation of apocrine gland cells should be controlled to a certain extent. When a somatic mutation has occurred in *BRCA1*, *BRCA2*,* p53*, or *p21*, however, it can lead to deleterious and unregulated proliferation of those cells F**(igure 5**).

## REGULATION OF *ABCC11* GENE Expression

In 2004, Bieche et al. (2004) reported that ABCC11 was up-regulated in estrogen receptor α-positive breast tumors, as compared with normal breast tissue. In contrast, Sosonkina et al. (2011) reported down-regulation of ABCC11 protein in human breast cancer. Park et al. (2006) investigated the mRNA levels of ABC transporter genes in breast cancer patients who underwent sequential weekly paclitaxel/FEC (5-fluorouracil, epirubicin, and cyclophosphamide) neoadjuvant chemotherapy. Their analysis showed that the expression of ABCC11 was increased (fold ratio = 2.71) in those patients with residual disease as compared with the patients having no pathologic evidence of any residual invasive cancer cells in the breast.

Honorat et al. (2008) have demonstrated that endogenous ABCC11 mRNA levels in breast cell lines are correlated with their estrogen receptor α-status. Interestingly, they found that ABCC11 expression was reduced *in vitro* by E_2_ treatments. Furthermore, this E_2_-dependent down-regulation of ABCC11 expression was blocked by co-treatment with tamoxifen, an E_2_ antagonist. These findings suggest that ABCC11 expression is regulated directly or indirectly by estrogen receptor α and that the prolonged exposure of breast cancer cells to tamoxifen can lead to up-regulation of ABCC11.

Hauswald et al. (2009) have shown that some of the histone deacetylase inhibitors induced the expression of several ABC transporters, including the *ABCC11* gene, to render acute myeloid leukemia cells resistant to a broad-spectrum of drugs. Molecular mechanisms underlying the induction remain to be elucidated. Since histone deacetylase inhibitors can be utilized in combination with conventional anti-cancer drugs in clinical trials, such induction of the ABCC11 WT may affect the efficacy of nucleoside-based chemotherapy.

## Relevance of ABCC11 Wt to Drug Resistance in Cancer Chemotherapy

The potential involvement of ABCC11 in drug resistance of breast cancer has recently been reported. For example, ABCC11 mRNA is found to be highly expressed in breast tumors (Bera et al., 2001; Yabuuchi et al., 2001; Bieche et al., 2004), and particularly in invasive ductal adenocarcinomas (available at: https://www.oncomine.org/resource/logn.html, accessed October 01, 2012). This expression is reportedly regulated by estrogen receptor-β (Honorat et al., 2008) and induced by 5-fluorouracil (5-FU; Oguri et al., 2007). Furthermore, it has been shown that ABCC11 is directly involved in 5-FU resistance by means of the efflux transport of the active metabolite 5-fluoro-2′-deoxyuridine 5′-monophosphate (FdUMP; Guo et al., 2003; Kruh et al., 2007; Oguri et al., 2007). It remains to be elucidated whether the expression of ABCC11 WT (538G) is related to drug resistance of breast cancer and high rates of mortality. Further clinical studies, including protein expression studies in tumors, will be needed to clarify the potential contribution of ABCC11 to breast cancer risk and prognosis, including drug resistance and chemosensitivity.

Because of their structural similarities, it could be anticipated that substrate specificity of ABCC11 would be related to those of ABCC4 and ABCC5. This indeed has been the case. Ectopic expression of ABCC11 in mammalian cells enhances the cellular efflux of cyclic nucleotides and confers resistance to certain anticancer and antiviral nucleotide analogs (Guo et al., 2003). In fact, it has been reported that ABCC11 WT has an ability to efflux cyclic nucleotides (e.g., cGMP and cAMP) and confers resistance to several antiviral and anticancer nucleotide analogs, such as 5′-FdUMP and 9′-(2′-phosohonylmethoxynyl)adenine (PMEA; Guo et al., 2003; Kruh et al., 2007; Oguri et al., 2007).

Therapy with nucleoside-derived drugs is characterized by inter-individual variability (Heinemann et al., 1988; Abbruzzese et al., 1991). Genetic variants that affect protein products involved in all steps leading to a drug’s action may be major contributors to this heterogeneity of responses to nucleoside-based treatments. In particular, variants of drug metabolizing enzymes and transporters might affect the amount of drug needed for an efficient therapeutic response (Errasti-Murugarren and Pastor-Anglada, 2010).

Successful treatment of cancer remains a therapeutic challenge, with a high percentage of patients suffering from drug resistance or relapsed disease. One of such examples involves anti-leukemia treatment with nucleoside analogs, such as cytarabine (AraC). Guo et al. (2009) have recently presented evidence that expression of ABCC11 WT is an important factor affecting acute myeloid leukemia patient survival. It is very likely that the cause of treatment failure in those patients with high expression of ABCC11 WT is an increased extrusion of AraC from blast cells mediated by the transporter.

Uemura et al. (2010) have recently found that both gene and protein expression of ABCC11 were higher in pemetrexed (MTA)-resistant cells than in the parental cells. The MTA-resistant cells showed cross-resistance to MTX, which is a substrate for ABCC11, and intracellular MTX accumulation in MTA-resistant cells was lower than that in the parental cells. They then tested the effect of decreasing the expression of ABCC11 by siRNA and found that decreased expression of ABCC11 enhanced MTA cytotoxicity and increased intracellular MTX accumulation in MTA-resistant cells. These findings suggest that ABCC11 confers resistance to MTA by enhancing the efflux of the intracellular anti-cancer drug.

They further analyzed the relationship between the *ABCC11* gene expression and MTA sensitivity of 13 adenocarcinoma cell lines. In contrast to their expectation, there was no correlation. Instead, the 13 lung adenocarcinoma cell lines could be classified into three groups based on the genotypes of the ABCC11 SNP (538G > A); G/G, G/A, and A/A. The A/A group showed a significant reduction in the IC_50_ value of MTA compared with those values for the combined G/G and G/A groups, indicating that *ABCC11* 538G > A is an important determinant of MTA sensitivity. These results suggest that *ABCC11* 538G > A may be one of the biomarkers for selection of MTA treatment in adenocarcinomas. This finding, however, should be carefully evaluated by clinical studies to determine whether *ABCC11* 538G > A is truly a clinically important biomarker for the prediction of chemotherapeutic efficacy.

## Conclusion

Apocrine secretion occurs when the secretory process is accomplished with a partial loss of cell cytoplasm. The secretory materials may be contained within the secretory vesicles or dissolved in the cytoplasm and then released during excretion as cytoplasmic fragments into the glandular lumen or interstitial space (Gesase and Satoh, 2003). Hitherto, apocrine secretory mechanisms have not been well characterized (Gesase and Satoh, 2003). Although the biochemical and physiological pathways that regulate the apocrine secretory process are not clearly known, our recent findings (Yoshiura et al., 2006; Toyoda et al., 2009; Inoue et al., 2010) that the SNP (538G > A, Gly180Arg) in the *ABCC11* gene determines both earwax type and axillary osmidrosis have shed light on the novel function of this ABC transporter in apocrine glands. Further studies are needed to explore the clinical significance of ABCC11 so as to elucidate whether there are any other diseases that involve apocrine secretion.

## Conflict of Interest Statement

The authors declare that the research was conducted in the absence of any commercial or financial relationships that could be construed as a potential conflict of interest.

## Figures and Tables

**Figure A2:**
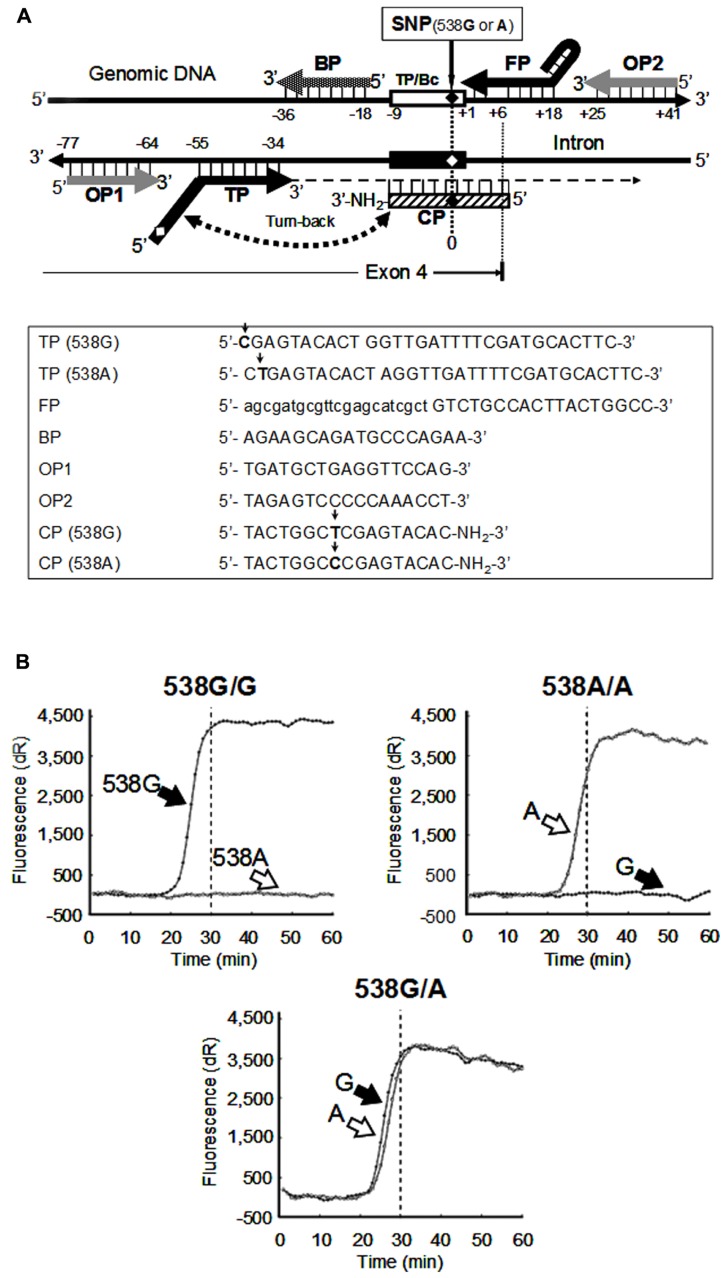
**Detection of SNP 538G A in *ABCC11* gene**. **(A)** Strategy and primers for the SmartAmp-based detection of SNP 538G > A residing in exon 4 of the *ABCC11* gene on chromosome 16q12 (upper panel) and the sequences of ABCC11 allele–specific primers needed for the SNP detection (lower panel). Arrows indicated the sequence difference between the WT and SNP alleles. **(B)** Time-courses of the SNP-detection reaction with ABCC11 allele-specific primers. Data are from Toyoda et al. (2009).

## Appendix

**Clinical Genotyping of SNP 538G A (Gly180Arg) in the *ABCC11* Gene**

The rapid growth of personalized medicine is being supported by emerging new technologies together with accumulating knowledge of pharmacogenomics. We tried to create a clinical method, the SmartAmp, to genotype the SNP 538G > A in the human *ABCC11* gene. The SmartAmp-based method enables us to detect genetic polymorphisms or mutations in about 30 min under isothermal conditions without requiring DNA isolation and PCR processes for sample preparation (Ishikawa and Hayashizaki, 2012). **Figure A2A** schematically illustrates the strategy of SNP detection by this clinical method. To determine the SNP 538G > A (Gly180Arg) in the *ABCC11* gene, we prepared one set of primers designated TP, FP, BP, OP, and CP (**Figure A2A**). The TPS discriminate the polymorphism 538G or 538A in the *ABCC11* gene, and the CPS inhibit the background amplification from mismatch sequence pairs (Toyoda et al., 2009; Ishikawa and Hayashizaki, 2012). These primers selectively recognized the SNP 538G > A of the *ABCC11* gene to discriminate homozygous 538G/G, heterozygous 538G/A, and homozygous 538A/A (**Figure A2A**). Thus, this genotyping method would provide a practical tool to support clinical diagnosis (Ishikawa and Hayashizaki, 2012).
